# Super-resolution deep learning reconstruction at coronary computed tomography angiography to evaluate the coronary arteries and in-stent lumen: an initial experience

**DOI:** 10.1186/s12880-023-01139-7

**Published:** 2023-10-30

**Authors:** Makoto Orii, Misato Sone, Takeshi Osaki, Yuta Ueyama, Takuya Chiba, Tadashi Sasaki, Kunihiro Yoshioka

**Affiliations:** 1 Department of Radiology, Iwate Medical University, 2-1-1, Idaidori, Yahaba, 028-3695 Japan; 2 Center for Radiological Science, Iwate Medical University, 2-1-1, Idaidori, Yahaba, 028-3695 Japan

**Keywords:** Coronary computed tomography angiography, Super-resolution deep learning reconstruction, Model-based iterative reconstruction, Signal-to-noise ratio, Contrast-to-noise ratio

## Abstract

A super-resolution deep learning reconstruction (SR-DLR) algorithm trained using data acquired on the ultrahigh spatial resolution computed tomography (UHRCT) has the potential to provide better image quality of coronary arteries on the whole-heart, single-rotation cardiac coverage on a 320-detector row CT scanner. However, the advantages of SR-DLR at coronary computed tomography angiography (CCTA) have not been fully investigated. The present study aimed to compare the image quality of the coronary arteries and in-stent lumen between SR-DLR and model-based iterative reconstruction (MBIR). We prospectively enrolled 70 patients (median age, 69 years; interquartile range [IQR], 59–75 years; 50 men) who underwent CCTA using a 320-detector row CT scanner between January and August 2022. The image noise in the ascending aorta, left atrium, and septal wall of the ventricle was measured, and the signal-to-noise ratio (SNR) and contrast-to-noise ratio (CNR) in the proximal coronary arteries were calculated. Of the twenty stents, stent strut thickness and luminal diameter were quantitatively evaluated. The image noise on SR-DLR was significantly lower than that on MBIR (median 22.1 HU; IQR, 19.3–24.9 HU vs. 27.4 HU; IQR, 24.2–31.2 HU, p < 0.01), whereas the SNR (median 16.3; IQR, 11.8–21.8 vs. 13.7; IQR, 9.9–18.4, p = 0.01) and CNR (median 24.4; IQR, 15.5–30.2 vs. 19.2; IQR, 14.1–23.2, p < 0.01) on SR-DLR were significantly higher than that on MBIR. Stent struts were significantly thinner (median, 0.68 mm; IQR, 0.61–0.78 mm vs. 0.81 mm; IQR, 0.72–0.96 mm, p < 0.01) and in-stent lumens were significantly larger (median, 1.84 mm; IQR, 1.65–2.26 mm vs. 1.52 mm; IQR, 1.28–2.25 mm, p < 0.01) on SR-DLR than on MBIR. Although further large-scale studies using invasive coronary angiography as the reference standard, comparative studies with UHRCT, and studies in more challenging population for CCTA are needed, this study’s initial experience with SR-DLR would improve the utility of CCTA in daily clinical practice due to the better image quality of the coronary arteries and in-stent lumen at CCTA compared with conventional MBIR.

## Introduction

Coronary computed tomography angiography (CCTA) is a robust noninvasive imaging technique with high spatial and temporal resolutions. Its diagnostic accuracy is high for the exclusion of coronary artery disease; however, some factors such as high image noise, insufficient vessel enhancement, and blooming- and beam-hardening artifacts may hamper the precise evaluation of vessel stenosis and in-stent lumen [1–3].

Recent CCTA developments with an ultrahigh spatial resolution computed tomography (UHRCT) of 0.25 mm × 128 or 160-row detector and model-based iterative reconstruction (MBIR) have overcome the limitations of the current CT spatial resolution. Several studies have reported higher diagnostic accuracy to detect significant stenosis with severely elevated calcium scores and in-stent patency with a diameter of ≥ 2.5 mm; however, the use of UHRCT is limited for patients with higher heart rate and weight and those with arrhythmia [4–6].

Recently, a super-resolution deep learning reconstruction (SR-DLR) algorithm trained using data acquired on the commercially available UHRCT system is available for the whole-heart, single-rotation cardiac coverage on a 320-detector row CT scanner [7, 8]. Several studies have reported the noise-reducing effect, improvement of spatial resolution, and the sharpness of margins and plaque detectability of coronary artery in SR-DLR compared with conventional reconstruction techniques [9, 10]. However, no studies have reported data on the potential value of SR-DLR at CCTA in the clinical setting. Thus, this study aimed to compare the image quality of the coronary arteries and in-stent lumen during CCTA reconstructed using SR-DLR and MBIR.

## Materials and methods

### Study population and design

Seventy consecutive patients who underwent CCTA for suspected or known coronary artery disease between January and August 2022 were prospectively enrolled at Iwate Medical University Hospital. Our exclusion criteria were renal insufficiency (estimated glomerular filtration rate < 30 mL/min per 1.73 m^2^), contrast agent allergy, history of bypass grafting, and potential pregnancy. Written informed consent was obtained from all participants, as approved by the institution’s human research committee.

### CT scanning

CT scans were performed using a 320-detector row CT scanner (Aquilion ONE PRISM Edition, Canon Medical Systems, Otawara, Japan). All patients were administered nitroglycerin. Patients with a heart rate of 65 bpm were administered oral beta-blockers 60 min before the CCTA scan based on the Society of Cardiovascular Computed Tomography guidelines [11, 12].

Coronary calcium scoring was performed using a prospective electrocardiogram (ECG) gating axial scan at a 75% RR interval, with 120 kV tube voltage and a 300-mA tube current. We delivered 25.9 mgI/kg/s of nonionic contrast material (iopamidol [Iopamiron 370, Bayer, Osaka, Japan]) at a 10-s fixed duration, followed by a 35-mL saline flush administered using a 20-G intravenous catheter. The scan parameters were collimation of 320 × 0.5 mm; rotation time, 0.275 s; z-coverage, 120–160 mm; tube voltage, 100 or 120 kV; and tube current, 180–750 mA. Prospective ECG gating scan with an acquisition window of 35–80% or 65–80% of the RR interval was used for patients with a heart rate of > 65 or ≤ 65 bpm, respectively. Retrospective ECG gating scan was used for patients with arrhythmia or those who could not sufficiently hold their breath. Experienced cardiovascular CT technologists determined the optimal stationary cardiac phase with minimum motion-free datasets. The dose length product was recorded for each participant, and the corresponding effective radiation dose was calculated using a standard conversion factor of 0.014 mSv/mGy cm for chest CT [13]. Axial images were reconstructed with 0.5-mm slice thickness and reconstruction interval. The image reconstruction field of view and the matrix size were 160–200 mm and 512 × 512, respectively. All images were reconstructed using the SR-DLR (PIQE) and MBIR algorithm (FIRST, Canon Medical Systems Corp.).

### Super-resolution deep learning reconstruction

**Fig. 1:**
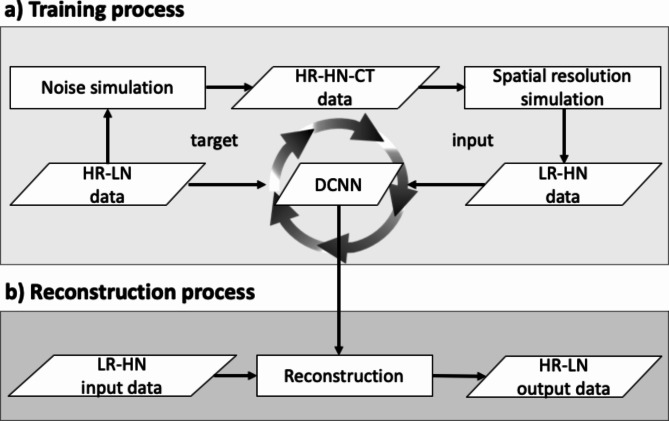
Training and reconstruction processing flow of super-resolution deep learning reconstruction. In training process **(a)**, the DCNN is trained by a lot of target and input pairs. The HR and LN data which are acquired on UHRCT scanner is used as target. The LR and HN data which is simulated from target is used as input. In reconstruction process **(b)**, the LR and HN data is reconstructed using the trained DCNN. Then the HR and LN data is obtained. DCNN, deep convolutional neural network; HR, high resolution; LN, low noise; UHRCT, ultrahigh spatial resolution computed tomography; LR, low resolution; HN, high noise

Super resolution technology aims at restoring high resolution information from low or normal resolution inputs. SR-DLR archived not only high spatial resolution but also noise reduction [8, 14]. SR-DLR uses deep learning technology. Figure 1 shows the SR-DLR processing flow. In training process (a), the deep convolutional neural network (DCNN) is trained by a lot of target and input pairs. The high resolution and low noise data which are acquired on UHRCT scanner is used as target. UHRCT equipped with a finer size detector and smaller x-ray focal spot source, provides diagnostic images with two times the spatial resolution compared to 320-detector row CT scanner. “Noise simulation” to simulate low-exposure scan and “Spatial resolution simulation” to simulate spatial resolution degradation are applied to the target data, and low-quality data with low spatial resolution and high noise content are used as input data. By learning these pairs of target and input data, DCNN can learn both the generation model of spatial resolution recovery and the noise model. In reconstruction process (b), by using the pre-trained DCNN, SR-DLR can reconstruct the image with high spatial resolution and low noise from low spatial resolution and high noise on 320-detector row CT scanner.

### Image interpretation

The image datasets were transferred to an off-line workstation, processed using commercially available software (Ziostation2, Ziosoft Inc., Tokyo, Japan) and two radiologists (M.O. and K.Y.) with 5 and 25 years of experience, respectively, in cardiovascular imaging performed all measurements. The readers were blinded to the clinical information and reconstruction method. In case of data analysis disagreed, a final decision was reached by consensus. The degree of coronary stenosis was graded as minimal (< 25%), mild (25–49%), moderate (50–69%), and severe (70–99%) [15].

The overall image quality of each coronary artery segment was rated based on a four-point rating score for each coronary artery segment (4, excellent [minimal or no noise-related blurring and diagnostic information sufficient); 3, good [some noise-related blurring and diagnostic information acceptable]; 2, fair [marked noise-related blurring and diagnostic information limited]; and 1, poor [blurry and diagnostic information impaired]) [16].

For SR-DLR and MBIR images, the image noise was recorded as the standard deviation (SD) of the attenuation value in a circular region of interest (ROI) placed in the ascending aorta, left atrium, and septal wall of the ventricle. Then, signal-to-noise ratio (SNR) was calculated as signal/noise in the proximal right coronary artery and left main trunk [6]. The contrast-to-noise ratio (CNR) was calculated as follows: (mean vessel lumen signal − mean perivascular fat signal)/image noise in the ascending aorta [17, 18]. ROI measurements were performed by two radiologists on axial images, carefully preventing calcifications, plaques, and stenosis.

### In-stent lumen assessability and quantitative stent analysis

**Fig. 2:**
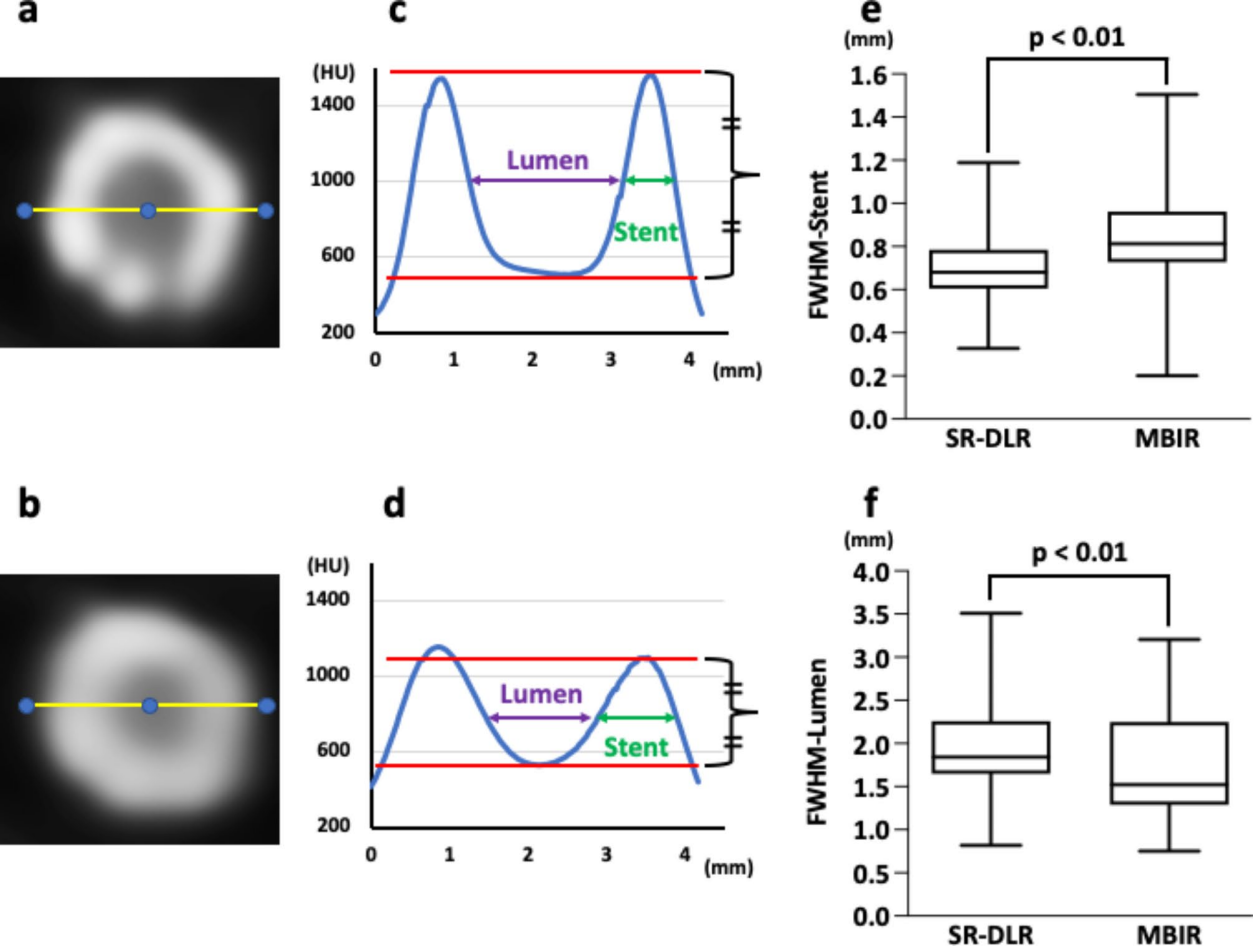
Quantitative analysis of the lumen and stent in the coronary artery. After identifying the center of the stent on a cross-sectional image, attenuation profiles were calculated (a: SR-DLR, b: MBIR). FWHM-lumen and FWHM-stent were measured (**c:** SR-DLR, **d:** MBIR). FWHM-stent was significantly thinner (p < 0.01), whereas FWHM-lumen was significantly larger on SR-DLR (p < 0.01) than on MBIR (**e**, **f**). SR-DLR, super-resolution deep learning reconstruction; MBIR, model-based iterative reconstruction; FWHM-lumen, full width at half maximum of the lumen; FWHM-stent, full width at half maximum of the stent

Stents were considered assessable with the absence of partial volume effects by stent struts, beam hardening, motion artifacts, calcification, or low CNR and when the lumen within the stent was clearly visible [4, 19]. Figure 2 shows the calculation methods of lumen visibility measurements using the average attenuation profile in the axial plane [4, 19]. First, the stent proximal and distal edges on the curved planar reformation (CPR) image were determined, with the first, second, and third quartiles as calculated points. Second, after determining the center of the stent on the cross-sectional image (Fig. 2a and b), attenuation profiles were calculated, and the full width at half maximum of the lumen (FWHM-lumen) and full width at half maximum of the strut (FWHM-stent) were measured (Fig. 2c and d).

### Invasive coronary angiography

Of the 70 patients, 14 underwent invasive coronary angiography. Experienced cardiovascular physicians performed selective invasive coronary angiography scanning using the standard Judkins technique, with a radial approach on a biplane angiography system (AlluraClarity FD10/10, Philips Electronics Japan, Tokyo Japan). Contrast medium was administered using an automated injection system (ACIST Cvi, ACIST Japan, Tokyo, Japan). The injection volume of contrast medium was 6.2 ml for the left coronary angiography, and 5.4 ml for the right coronary angiography.

### Statistical analysis

Statistical analyses were performed using IBM® SPSS® 28.0.1 (IBM Corporation., Armonk, NY, USA). Continuous measurements are expressed as mean ± SD for normally distributed variables or median (interquartile range [IQR], 25th–75th percentile) for nonparametric data and compared using Student’s t-test or Mann–Whitney U-test as appropriate. Categorical variables are expressed as numbers and percentages. p-value of < 0.05 was considered statistically significant. The interobserver agreement between two radiologists regarding the qualitative evaluation was evaluated using the Cohen kappa κ coefficient. A κ value of more than 0.81 corresponded to excellent interobserver agreement, while values of 0.61–0.80 corresponded to good agreement.

## Results

Table 1 lists the baseline clinical characteristics of 70 patients (median age, 69 (IQR, 59–75) years; 50 men). Fifteen patients had prior stent placement (a total of 20 stents). The median body mass index of patients was 24 (IQR, 22–26) kg/m^2^; their median heart rate during scanning was 55 (IQR, 51–61) bpm.

**Table 1:** Baseline characteristics

Patient demographics (n = 70)	
Age, years	69 (59–75)
Male patients, n (%)	50 (71)
Height, cm	166 (159–171)
Weight, kg	65 (56–73)
Body surface area, m^2^	1.7 (1.6–1.9)
Body mass index, kg/m^2^	24 (22–26)
Hypertension, n (%)	48 (69)
Dyslipidemia, n (%)	39 (56)
Diabetes, n (%)	22 (31)
Smoking history (former or current), n (%)	18 (25)
Sinus rhythm, n (%)	65 (93)
Characteristics of CCTA scanning	
Mean heart rate during CCTA scanning, bpm	55 (51–61)
Tube voltage 100/120 kV, n (%)	32 (45)/38 (55)
Tube current	
With 100 kV, mA	315 (250–368)
With 120 kV, mA	400 (260–565)
Total dose length product, mGy*cm	62 (44–117)
Total effective radiation dose, mSv	0.9 (0.6–1.6)
Contrast medium dose, mL	48 (42–58)
Calcium score	16 (0–180)

CCTA, coronary computed tomography angiography

Of the 70 patients, 63 underwent imaging with prospective ECG gating, and the other seven were imaged with retrospective gating due to arrhythmia or insufficient breath-hold. In patients without stents, the median calcium score was 16 (IQR, 0–180). The median effective radiation dose was 0.9 (IQR, 0.6–1.6) mSv.

### Comparison of SR-DLR and MBIR

Table 2displays the reconstruction time, image noise, SNR, CNR, and visual evaluation of the coronary arteries between SR-DLR and MBIR. The reconstruction time was significantly shorter on SR-DLR than on MBIR (median, 97 s; IQR, 88–109 s vs. 173 s; IQR, 164–187 s, p < 0.01). The image noise was significantly lower on SR-DLR than that on MBIR (median 22.1 HU; IQR, 19.3–24.9 HU vs. 27.4 HU; IQR, 24.2–31.2 HU, p < 0.01). The SNR (median 16.3; IQR, 11.8–21.8 vs. 13.7; IQR, 9.9–18.4 HU, p = 0.01) and CNR (median 24.4; IQR, 15.5–30.2 vs. 19.2; IQR, 14.1–23.2, p < 0.01) on SR-DLR was significantly higher than those on MBIR. The image quality score on SR-DLR was significantly higher than those on MBIR (median 4.0; IQR, 4.0–4.0 vs. 3.0; IQR, 3.0–4.0, p < 0.01).

**Table 2:** Comparison of reconstruction time, image noise, signal-to-noise ratio, contrast-to-noise ratio, and visual evaluation of coronary arteries between super-resolution deep learning reconstruction and model-based iterative reconstruction

Parameter	SR-DLR	MBIR	p-value
Reconstruction time, s	97 (88–109)	173 (164–187)	< 0.01
Image noise, HU			
Ascending aorta	22.5 (20.5–31.8)	29.1 (26.2–36.6)	< 0.01
Left atrium	22.4 ± 4.0	30.1 ± 5.4	< 0.01
Septal wall of the ventricle	20.5 ± 3.6	23.7 ± 3.8	< 0.01
All locations	22.1 (19.3–24.9)	27.4 (24.2–31.2)	< 0.01
SNR	16.3 (11.8–21.8)	13.7 (9.9–18.4)	0.01
CNR	24.4 (15.5–30.2)	19.2 (14.1–23.2)	< 0.01
Overall quality (scale 1–4)	4.0 (4.0–4.0)	3.0 (3.0–4.0)	< 0.01

SR-DLR, super-resolution deep learning reconstruction; MBIR, model-based iterative reconstruction; SNR, signal-to-noise ratio; CNR, contrast-to-noise ratio

**Fig. 3:**
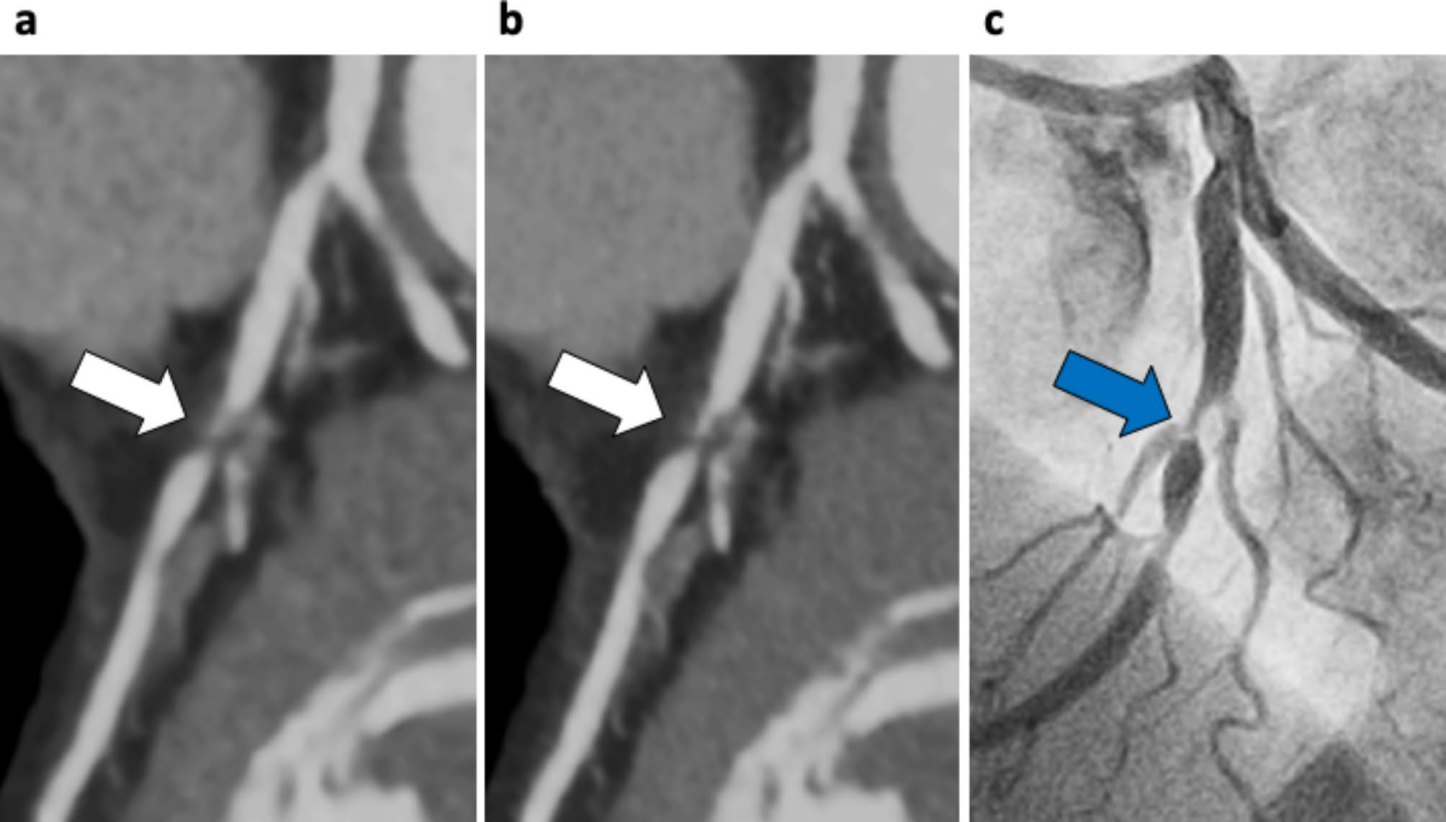
Case example of a 66-year-old woman with effort angina. Curved planar reformation of the left anterior descending artery (LAD) on MBIR **(a)** and SR-DLR **(b)**. Severe stenosis in the proximal section of the LAD was more sharply delineated on SR-DLR than those on MBIR (white arrows), which was confirmed using invasive coronary angiography (**c**, blue arrow). SR-DLR, super-resolution deep learning reconstruction; MBIR, model-based iterative reconstruction; LAD, left anterior descending artery

**Fig. 4:**
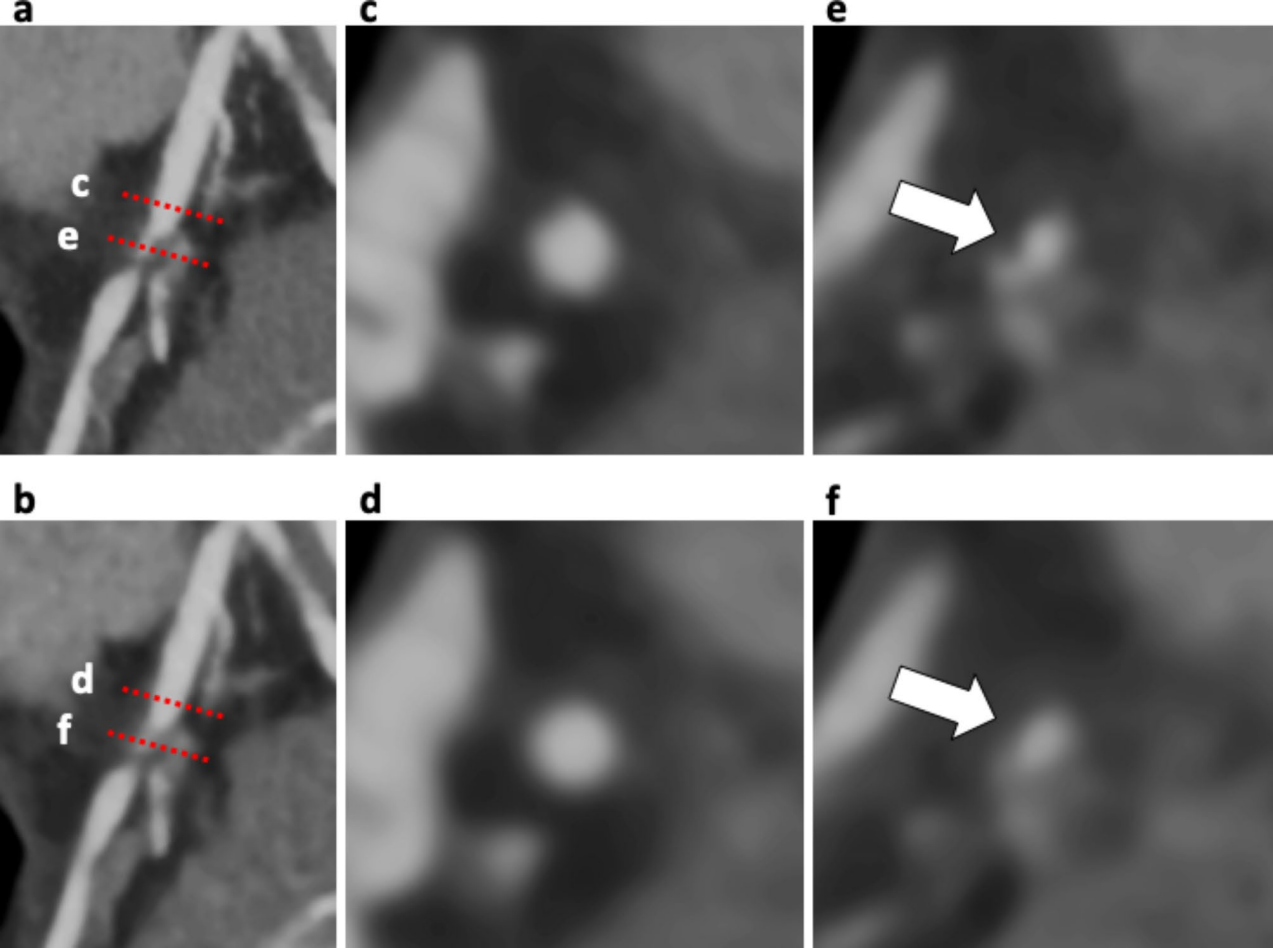
Case example of the patient in Fig. 2. Curved planar reformation (CPR) images in the proximal section of the left anterior descending artery (LAD) with SR-DLR **(a)** and MBIR **(b)**. Two cross-sectional images show the proximal reference site (**c** and **d**) and the site of maximal stenosis (**e** and **f**) correspond to red dashed lines on the CPR image. Coronary lumen and plaque (white arrows) were more sharply delineated on SR-DLR (**c**, **e**) than those on MBIR (**d**, **f**). CPR, curved planar reformation; LAD, left anterior descending artery; SR-DLR, super-resolution deep learning reconstruction; MBIR, model-based iterative reconstruction

**Fig. 5:**
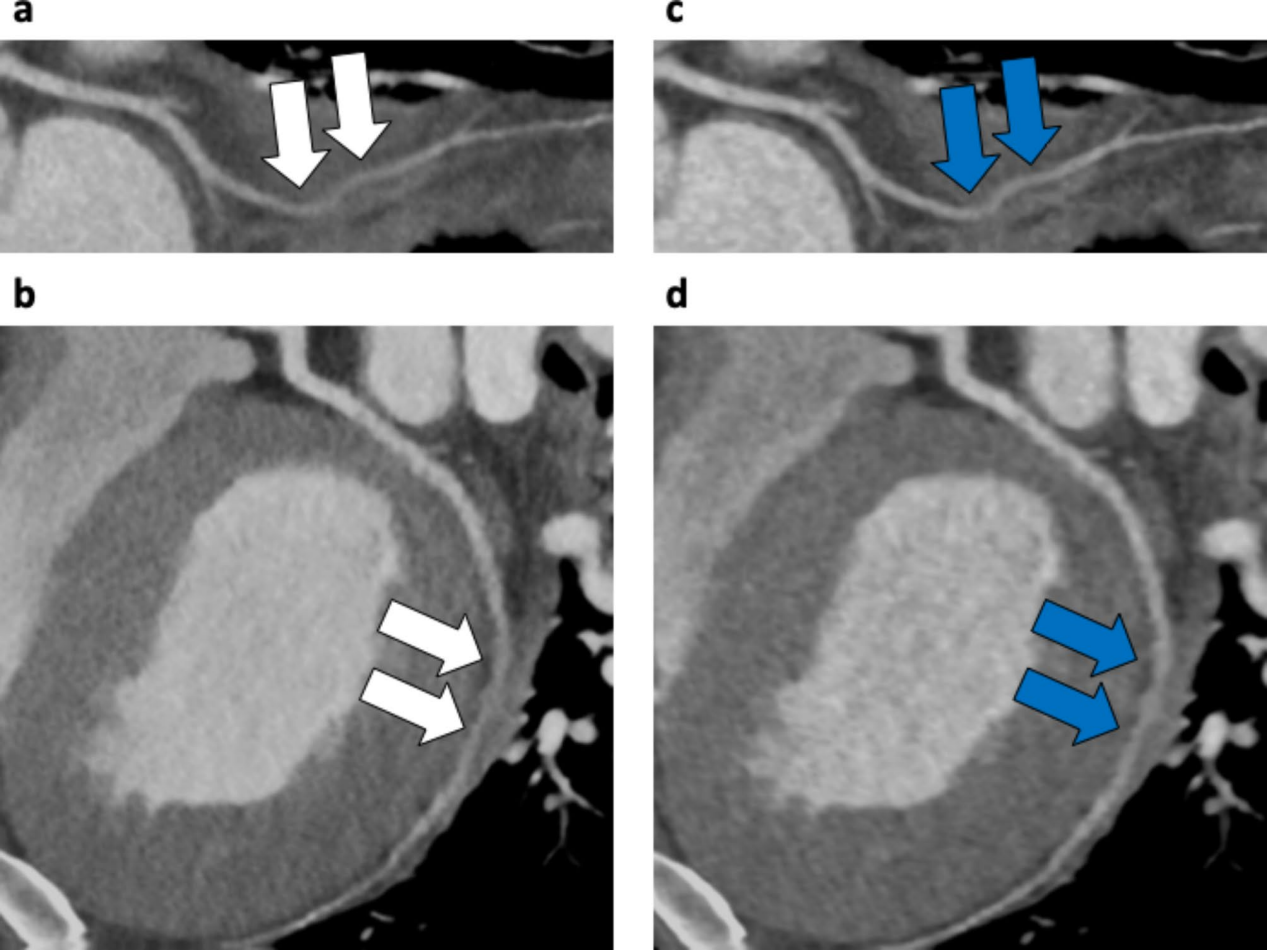
Case example of a 34-year-old man weighing 144 kg with a body mass index of 46 kg/m^2^. Curved planar reformation of the left circumflex artery taken at 120 kV, 750 mA, with prospective ECG gating. **a**, **b**: SR-DLR image (signal-to-noise ratio (SNR), 17.4; contrast-to-noise ratio (CNR), 21.0). The image quality was rated as fair with marked noise-related blurring (white arrows). **c**, **d**: MBIR image (SNR, 16.0; CNR, 17.8). The image quality was rated as good with some noise-related blurring (blue arrows). SR-DLR, super-resolution deep learning reconstruction; SNR, signal-to-noise ratio; CNR, contrast-to-noise ratio; MBIR, model-based iterative reconstruction

In both two-volume datasets, a total of 210 major coronary branches were evaluated (Table 3), and representative cases are shown in Figs. 3 and 4. Severe stenosis in the proximal section of the left anterior descending artery was more sharply delineated on SR-DLR than on MBIR, as confirmed by invasive coronary angiography. In contrast, the image quality was lower on SR-DLR images than those on MBIR in patients weighing 144 kg with a body mass index of 46 kg/m^2^ even with higher SNR and CNR on SR-DLR images (Fig. 5). A substantial interobserver agreement was observed on the overall image quality (κ = 0.83).

**Table 3:** Characteristics of coronary artery stenosis and stent

Stenosis assessment (n = 210)	
<25%, n (%)	75 (36)
25–49%, n (%)	82 (39)
50–69%, n (%)	36 (17)
70–99%, n (%)	17 (8)
Stent diameter (n = 20)	
2.25 mm, n (%)	1 (5)
2.5 mm, n (%)	7 (35)
2.75 mm, n (%)	2 (10)
3.0 mm, n (%)	7 (35)
3.5 mm, n (%)	2 (10)
4.0 mm, n (%)	1 (5)
Stent length (n = 20)	27 ± 11
Stent lesion (n = 20)	
Right coronary artery, n (%)	3 (15)
Left anterior descending artery, n (%)	12 (60)
Left circumflex artery, n (%)	5 (25)

### Coronary stent analysis

**Fig. 6:**
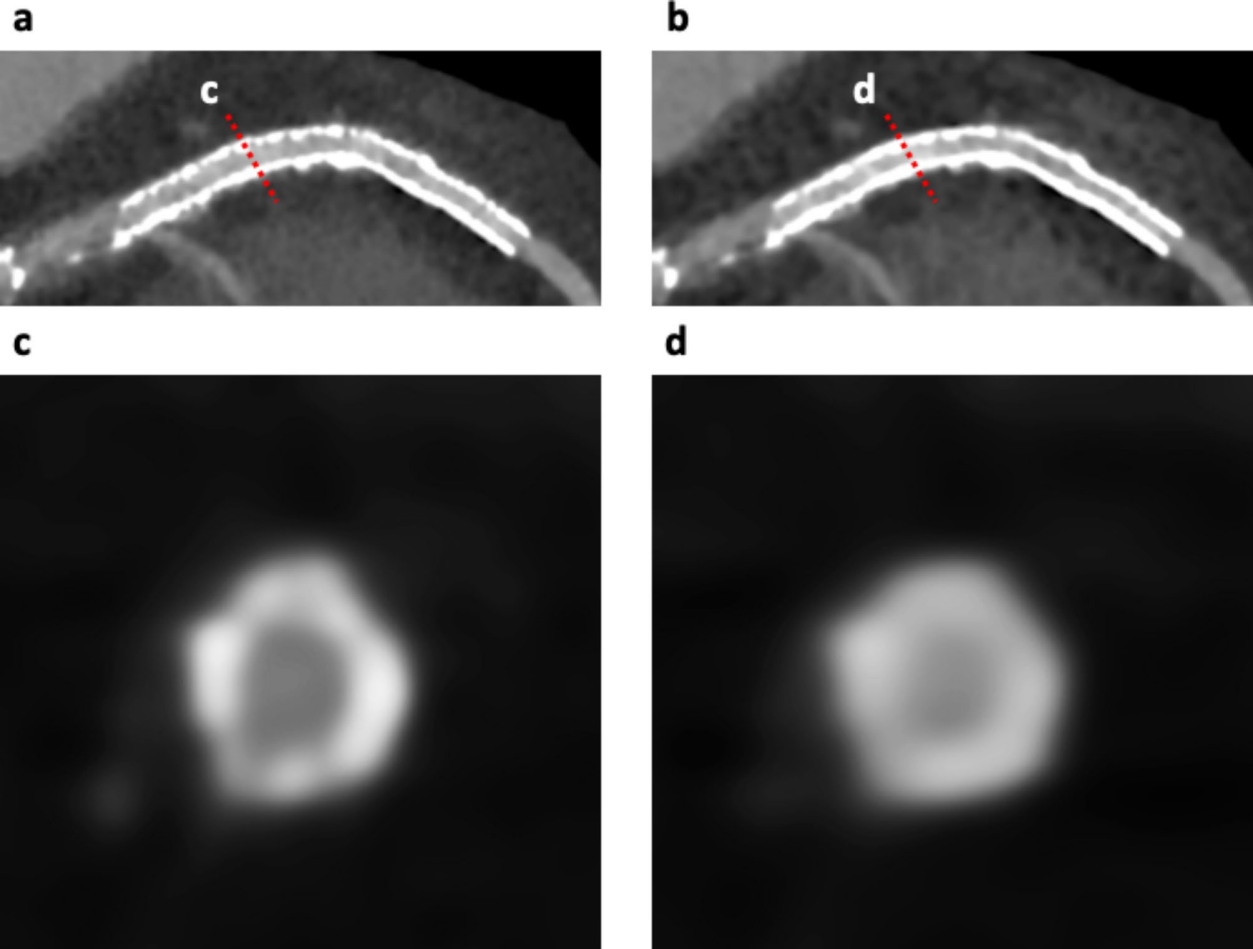
Case example of an 80-year-old woman with a stent in the left descending coronary artery. Curved planar reformation (CPR) images of the 2.5 mm stent in the left anterior descending artery (LAD) with SR-DLR **(a)** and MBIR images **(b)**. Cross-sectional images showing the in-stent lumen and stent strut (**c** and **d**) corresponding to red dashed lines on the CPR image. In-stent lumen was clearly visualized on SR-DLR (**a** and **c**; assessable) compared with MBIR (**b** and **d**; unassessable). CPR, curved planar reformation; LAD, left anterior descending artery; SR-DLR, super-resolution deep learning reconstruction; MBIR, model-based iterative reconstruction

In 20 stents from fifteen patients, stent assessability was compared between SR-DLR and MBIR (Table 3; Fig. 6). On SR-DLR, stent assessability was 100% (1, 7, 2, 7, 2, and 1 of 2.25, 2.5, 2.75, 3.0, 3.5, and 4.0 mm stents). On MBIR, 2.25 mm stents and 3/7 of the 2.5 mm stents cannot be assessed, and the remaining sixteen stents can be assessed (four stents unassessable on MBIR could be assessed on SR-DLR). All twelve stents with a diameter greater than 2.75 mm were assessable both on SR-DLR and MBIR. On quantitative analysis, 60 axial slices of 20 stents were evaluated. The FWHM-stent on SR-DLR was significantly thinner than on MBIR (median, 0.68 mm; IQR, 0.61–0.78 mm vs. 0.81 mm; IQR, 0.72–0.96 mm, p < 0.01; Fig. 2e). The FWHM-lumen calculated on SR-DLR was significantly larger than on MBIR (median, 1.84 mm; IQR, 1.65–2.26 mm vs. 1.52 mm; IQR, 1.28–2.25 mm, p < 0.01; Fig. 2f).

## Discussion

To the best of our knowledge, this is the first study to evaluate the SR-DLR on the image quality of the coronary arteries and in-stent lumen. The image noise was significantly lower, and the SNR and CNR at the proximal coronary arteries was significantly higher on SR-DLR than those on MBIR images. Thus, the overall image quality was significantly better on SR-DLR images. Furthermore, stent struts were significantly thinner, and in-stent lumens were significantly larger on SR-DLR than on MBIR in coronary stent analysis.

### Development of CCTA

CCTA has become a first-line test to evaluate patients suspected of coronary artery disease. Nevertheless, some factors such as high image noise, insufficient vessel enhancement, and blooming- and beam-hardening artifacts may hamper the precise evaluation of vessel stenosis and stents [1–3]. Recent developments of UHRCT have enabled the evaluation of calcified, stented, or small diameter segments due to the greater spatial resolution [4–6]. UHR of CCTA with dual-source photon-counting detector CT have also enabled the visualization of calcified plaques with an excellent spatial resolution [20]. However, radiation exposure for UHRCT was higher than that for conventional CCTA [5]. Moreover, UHRCT cannot be used in patients with higher heart rates and weight and those with arrhythmia due to the limitation of the gantry rotation time [4–6]. Remarkably, the whole heart coverage by the area detector with a faster rotation speed contributed to the improvement in the temporal resolution and decreased the radiation exposure and contrast media.

### SR-DLR performance

Recently, DLR was developed to improve spatial resolution and low-contrast detectability while reducing noise due to the power of machine learning, a form of artificial intelligence [16]. Especially, SR-DLR algorithm trained using data acquired on the commercially available UHRCT system is available [7, 8]. SR-DLR algorithm features UHR 0.25 mm detectors to maximize the inherent spatial resolution on the conventional 320-detector row CT. With conventional reconstruction, a long tradeoff between spatial resolution and noise has forced CT systems to operate at much lower spatial resolution levels than the detector and focal spots are capable of producing maintenance at clinically acceptable dose levels. With DLR, not only can this resolution be utilized, without increasing the dose or noise, but it also can be enhanced further. Furthermore, the neural network behind SR-DLR does not learn features solely in the axial plane but rather in three dimensions, indicating that signal features are identified and preserved in all three planes. Therefore, SR-DLR is found to be well-suited for cardiac exams, which are usually reviewed in CPR.

In several studies, the spatial resolution is reported be higher on MBIR than on filtered back projection and hybrid-IR images [21, 22]. Reportedly, the sophisticated MBIR modeling reduces blooming artifacts and yields a better image quality than hybrid IR [23]. Recently, several studies have reported that the image noise was lower and the coronary artery delineation was better on SR-DLR scans compared with conventional reconstruction techniques such as hybrid-IR, MBIR, and DLR [9, 10]. In this study, SR-DLR images yielded a higher image quality including image noise reduction and better spatial resolution than those on MBIR. Moreover, the reconstruction time was significantly shorter on SR-DLR than on MBIR. Hence, using SR-DLR would improve the utility of CCTA in routine clinical practice.

### Assessment of the in-stent lumen on CCTA with SR-DLR

Direct visualization of the in-stent lumen on CCTA was challenging mainly due to beam hardening and partial volume effects. Motoyama et al. have reported that the in-stent lumen of stents with ≥ 2.5 mm diameter could be assessed on UHRCT because of improved spiral resolution [4]. In this study, SR-DLR could evaluate all stents, including 2.25 and 2.5 mm stents. Moreover, SR-DLR demonstrates better strut delineation of the stent and in-stent lumen that are more accurate than MBIR. The edge has been reportedly sharper on MBIR than on images reconstructed with conventional filtered back projection or hybrid IR [23, 24]. SR-DLR had similar effects on the boundary of coronary stents.

In contrast, the image quality of SR-DLR was lower in patients with higher weight. Training the deep learning neural network with educational images from higher weight patients would make the SR-DLR more valuable.

### Limitations

First, our study population was small. Second, the diagnostic accuracy of CCTA was confirmed in comparison with invasive coronary angiography in a limited number of enrolled patients only. Large-scale studies are required to validate and expand our initial experience using invasive coronary angiography as the reference standard. Third, the SNR and CNR were evaluated in the proximal coronary arteries only. The small diameter of distal segments makes it impossible to place ROIs without including parts of the vessel walls and adjacent tissues. Moreover, no stents ware evaluated on side-branch in this study. SR-DLR may be able to evaluate side-branch stents as well as small-diameter stents based on the results of this study, but further studies are needed to evaluate side-branch stents. Forth, the SR-DLR algorithm is currently vendor-specific. Finally, the population of this study is less challenging for CCTA scans. In further studies, the value of SR-DLR needs to be proven in patients with high calcium scores to assess the delineation of the calcified plaques, in patients with high body weight, and in patients with arrhythmias. Comparative studies of coronary stent evaluation, including stents in side-branch, between UHRCT and 320-detector row CT scanner with SR-DLR are also required to assess whether SR-DLR can overcome the limitations of UHRCT.

## Conclusion

SR-DLR might improves the image quality of the coronary arteries and in-stent lumen at CCTA. Datasets reconstructed with SR-DLR empower the clinician with the high-contrast signal definition and reduce noise, relative to conventional MBIR.

## Data Availability

The dataset analyzed during the present study is available from the corresponding author on reasonable request.

## Abbreviations

CCTA: Coronary computed tomography angiography
SR-DLR: Super-resolution deep learning reconstruction
MBIR: Model-based iterative reconstruction
UHRCT: Ultrahigh spatial resolution computed tomography
ECG: Electrocardiogram
DCNN: Deep convolutional neural network
SD: Standard deviation
ROI: Region of interest
SNR: Signal-to-noise ratio
CNR: Contrast-to-noise ratio
CPR: Curved planar reformation
FWHM-lumen: Full width at half maximum of the lumen
FWHM-stent: Full width at half maximum of the stent
IQR: Interquartile range
HR: High resolution
LR: Low resolution
HN: High noise
LN: Low noise
